# Mindfulness Practice with a Brain-Sensing Device Improved Cognitive Functioning of Elementary School Children: An Exploratory Pilot Study

**DOI:** 10.3390/brainsci12010103

**Published:** 2022-01-12

**Authors:** Boglarka Vekety, Alexander Logemann, Zsofia K. Takacs

**Affiliations:** 1 Doctoral School of Education, Faculty of Education and Psychology, ELTE Eötvös Loránd University, 1075 Budapest, Hungary; vekety.boglarka@ppk.elte.hu; 2 MTA-ELTE Lendület Adaptation Research Group, 1064 Budapest, Hungary; 3 Institute of Psychology, Faculty of Education and Psychology, ELTE Eötvös Loránd University, 1064 Budapest, Hungary; alexander.logemann@ppk.elte.hu; 4 Clinical Psychology, School of Health in Social Science, University of Edinburgh, Edinburgh EH8 9AG, UK

**Keywords:** mindfulness training, EEG-feedback, neurofeedback, brain-sensing device, brain–computer interface (BCI), executive functions, children, technology

## Abstract

This is the first pilot study with children that has assessed the effects of a brain–computer interface-assisted mindfulness program on neural mechanisms and associated cognitive performance. The participants were 31 children aged 9–10 years who were randomly assigned to either an eight-session mindfulness training with EEG-feedback or a passive control group. Mindfulness-related brain activity was measured during the training, while cognitive tests and resting-state brain activity were measured pre- and post-test. The within-group measurement of calm/focused brain states and mind-wandering revealed a significant linear change. Significant positive changes were detected in children’s inhibition, information processing, and resting-state brain activity (alpha, theta) compared to the control group. Elevated baseline alpha activity was associated with less reactivity in reaction time on a cognitive test. Our exploratory findings show some preliminary support for a potential executive function-enhancing effect of mindfulness supplemented with EEG-feedback, which may have some important implications for children’s self-regulated learning and academic achievement.

## 1. Introduction

It is difficult to overestimate the role of children’s self-regulatory skills and attention regulation in education. In fact, self-regulation and executive functions (inhibition, working memory, and cognitive flexibility) are strong predictors of children’s academic achievement [1,2,3]. As prior meta-analyses have shown, one of the most effective ways to enhance executive functions and self-regulation has been proven to be mindfulness-based interventions [4,5,6,7]. Mastering the skills related to mindfulness can facilitate learners’ self-awareness to recognize moments of mind-wandering and practice self-regulation by redirecting attention to the here-and-now from task-irrelevant thoughts [8]. However, practicing mindful attention can be especially difficult for children because there are no overt signs of awareness that can be used for feedback by the teacher. In that vein, providing scaffolding through feedback on the electrical activity of the brain, which is known to vary as a function of mindful awareness, may assist the learning process and facilitate the effects of mindfulness practices [9,10]. Moreover, supplementing mindfulness with a brain-sensing device can empower children to regulate their own attention, which in turn could lead to self-regulated or mindful learning [11,12].

Despite increasing evidence for the benefits of mindfulness with electroencephalographic (EEG) feedback on adults’ attention and psychological outcomes [13,14,15,16,17,18,19], its effects in children are less studied. Two available studies with elementary school children found that mindfulness practice with EEG-feedback successfully improved subjective measures of attention and discipline reported by teachers [20,21]. The present study aimed to extend these prior investigations by examining the effects of mindfulness training supplemented with EEG-feedback on objective measures of executive functions and brain activity correlates among typically developing elementary school children. The significance of this also lies within the fact that learning environments have been undergoing a fundamental change in the last decade driven by the widespread availability of digital technology and the intention to empower children to promote their own mental health and learning [22,23]. In that vein, the current study has important relevance for educational practice and might serve, in addition to providing implications in the applied context, as a guide for future research that plans to investigate such technology combined with mindfulness within an educational context.

### 1.1. Mindfulness-Related Skills and Brain Mechanisms

In recent decades, the concept of mindfulness defined by Jon Kabat-Zinn [24] as ‘the awareness that emerges through attention on purpose in the present moment with a nonjudgmental attitude’ has been supplemented by cognitive theories that describe mindfulness as a ‘special’ attentional state [25] and as a ‘set of neurocognitive-behavioral skills’ that cultivate the regulation of attention and executive functions [26]. Many previous researchers have agreed upon the central role of the voluntary control of attention in the development of mindfulness, which facilitates the effects on cognitive, behavioral, and affective skills, including metacognition and executive functions [27,28,29,30,31]. Executive functions (EFs) are defined as top-down regulated, goal-directed cognitive processes involved in monitoring and controlling one’s behavior [32]. Poor EFs, such as the inability to regulate attention, delay gratification, and flexibly switch between cognitions or behaviors to solve problems, have been associated with a host of short- and long-term problems across the lifespan, including school failure, drug abuse, and psychological disorders [3,31]. However, previous research has demonstrated that EFs can be enhanced, and they are especially malleable in childhood; thus, early interventions that address these skills are of enormous relevance [33]. The pedagogical relevance of nurturing EFs in schools is further supported by findings from previous decades that showed teachers reported 15 to 50% percent of children to have behavior problems related to EFs, such as paying attention, remembering instructions, completing tasks independently, transitioning between tasks, or controlling automatic responses, such as raising their hand before participating or taking turns [34,35]. As a prior meta-analysis investigating the effects of interventions in childhood pointed out, mindfulness is one of the most effective interventions to enhance EFs among all the different behavioral approaches [5].

Mindfulness-induced changes in self-regulation have been associated with neuropsychological mechanisms approached by EEG studies investigating neural oscillations [34,35,36]. Neural oscillations serve as key mechanisms in enabling communication between distant brain areas, with oscillations of different frequencies corresponding to different brain network configurations and processes [37]. Accordingly, prior research has mainly reported alpha (8–12 Hz) and theta (4–8 Hz) oscillations as brain activity correlates of mindful awareness, with an acute increase in amplitude during mindfulness practices and in the resting state after training [34,35,38,39]. Alpha brain activity has been reported to play a role in attention regulation, inhibition, information processing, and filtering incoming sensory input from the environment [27,39,40,41]. Similar to alpha oscillations, theta oscillations have also been associated with inhibitory processes, in addition to memory consolidation processes; however, they also signal a deep internally focused state connected to self-reflection [27]. In contrast, low beta brain activity has been associated with external attention in a wakeful state, while increased high beta activity (22–30 Hz) has been related to arousal and stress [42,43].

Based on these findings from neuropsychology and the recent development of brain–computer interfaces (BCIs), EEG-feedback protocols have been composed to target mindfulness-related neuromodulation. This application of EEG-feedback is strikingly different from classical neurofeedback to reduce attention-deficit/hyperactivity disorder (ADHD) symptomatology. Generally, EEG-feedback protocols to reduce ADHD symptoms suppress theta activity and enhance low beta activity (12–20 Hz) [44]. In contrast, EEG-feedback protocols for mindfulness-related brain activity aim to reinforce activity in alpha and theta bands with positive feedback while decreasing high beta activity [45,46,47,48]. Additionally, studies of mindfulness training and alpha-based neurofeedback training have found that both can lead to increases in alpha power and mindful awareness, and have proposed alpha as a mediator of the training effect on cognitive functioning [45,46,49,50,51,52,53,54]. This improvement in regulating alpha waves exerts its positive influence on learning by allocating attentional resources more fully during early processing phases and by the neuromodulation of beta waves to slower alpha waves, the process of which is responsible for memory consolidation [29,55].

### 1.2. Previous Studies on Mindfulness with a Brain-Sensing Device

The number of studies investigating the effect of mindfulness training with EEG-feedback for adults has been constantly growing in this decade, showing positive effects on attention and cognitive performance [14,16,18,47] and a relaxed mindset with reduced stress and anxiety when compared to a control condition [15,17,46,47]. However, evidence from other research regarding mindfulness with EEG-feedback has not shown a conclusive positive effect; thus, it is still an open question whether such technological innovations can render any positive effects and support traditional mindfulness training [13,19,52].

In a prior study, Bhayee and colleagues [16] randomly assigned adult participants to either real-time mindfulness training with auditory EEG-feedback with a wearable Muse brain-sensing headband and mobile application for 6 weeks or an active control group with the same amount of time solving online math problems. The results revealed that participants in the intervention group had a significantly larger reduction in reaction time from pre- to post-test on the Stroop task than the active control group, which may suggest that the mindfulness group’s inhibitory responses and attention became faster and thus more efficient. Likewise, Crivelli and colleagues [17] implemented 4 weeks of intensive mindfulness training with auditory EEG-feedback supported by the Muse brain-sensing device. Adult participants were randomly allocated to either the neurofeedback-assisted mindfulness meditation group or the active control group. The mindfulness group demonstrated a relaxed mindset, improved electrophysiological markers of attention regulation (i.e., alpha/beta ratio at resting-state, event-related potentials during a Stroop-like task) and improved cognitive performance as measured by a complex reaction time task. Another prior study by Hunkin and colleagues [47] found that BCI-assisted mindfulness training with a Muse headband resulted in an increased state of mindfulness and less mind-wandering than the training with regular mindfulness (without feedback) group. Interestingly, they also found that for some participants, receiving feedback on mind-wandering during mindfulness practice heightened their arousal and frustration; thus, for them, the feedback was rather distracting and incongruent with their subjective experience. These studies with adult participants provide some initial support for the effect of supplementing mindfulness practice with EEG-feedback to improve aspects of attention and EFs; however, it is much less studied whether this novel approach is feasible and effective with children. To the best of our knowledge, there have been only two empirical studies with children; however, both of these studies measured the effects using only subjective reports by teachers. A study by Martinez and Zhao [21] found that 3 min of technology-supported mindfulness practice with the Muse headband once a week for 6 months resulted in fewer office discipline referrals among 13- to 14-year-old students. Another empirical study from Antle and colleagues [20] implemented 24 sessions of mindfulness training with an alpha and theta visual EEG-feedback protocol to reduce anxiety and increase mindful attention. Computer games were combined with visual feedback to create an age-appropriate environment for 5- to 11-year-old children. According to their findings, this protocol successfully increased within-group relaxation and mindful attention from pre- to post-test in a classroom context, as reported by teachers and school counselors. Lastly, the study of Mishra and colleagues [56] explored the effects of a closed-loop digital meditation intervention (without EEG-feedback), with performance-adaptive and adjusted task difficulty, on the neurocognitive functioning of 10–18 year old adolescents who experienced early childhood neglect. They found a significant improvement in neural functional connectivity in the domains of cognition and hyperactive behavior as well as higher academic performance in the digital meditation group. Although these results are promising, no studies have been conducted assessing the effects of mindfulness with EEG-feedback on objective measures of executive functions and brain activity correlates compared to a control condition. The current study extends the prior research by assessing whether mindfulness supplemented with EEG-feedback modulates plausible mindfulness-related electrophysiological correlates and translates to observable benefits in terms of objective measures of executive functions, which are essential skills for academic performance and self-regulated learning.

### 1.3. Hypotheses

In particular, we expected that: (i) children in the mindfulness with auditory EEG-feedback group would show systematically increased slow neural oscillatory brain activity (specifically alpha and theta) during the eight sessions, mirrored by the calm/focused and active/mind-wandering states as logged by the Muse application; (ii) the mindfulness with EEG-feedback group would show increased theta and alpha amplitude from pre- to post-test at resting-state compared to the control group; and (iii) neuromodulation would be accompanied by improvement from pre- to post-test on measures of executive functions regarding accuracy and reaction time compared to the control group.

## 2. Materials and Methods

### 2.1. Sample

Children who were 8 to 12 years old were recruited from a local primary school in Budapest, Hungary (2019). From the six classes within this age range, two head teachers of fourth grade classes were willing to participate in the research program. After a verbal presentation about the research, an information letter and written consent were provided for all parents. Children with a diagnosis of a psychological disorder were excluded from participating in the study. From the two fourth grade classes, 31 parents applied to participate in the research. Using G-power, we determined that a total sample size of at least 22 participants would suffice to detect between/within-factor interactions, assuming a moderate effect size (0.09 partial eta squared) with alpha set at 0.05 and power at 0.8. The participants were from families of middle and high socioeconomic status. The gender distribution was relatively close to equal within the whole group, with 51% girls and 49% boys. The age range was 9–10 years (*M* = 9.92; *SD* = 4.35). None of the participants dropped out.

### 2.2. Research Design

This exploratory pilot study was a 4 week randomized controlled trial (RCT). To assess the efficacy of mindfulness training with EEG-feedback on EFs and neural oscillations, the intervention group was compared to a passive control group (with no treatment, only pre- and post-test). After the pretest, children were matched by their age, gender, and executive function pretest scores to relatively equal pairs, then randomly allocated with a random number generator to either the mindfulness group with EEG-feedback or the passive control group.

### 2.3. Measures

#### 2.3.1. Location–Direction Stroop-Like Arrow Test

In this computer-based neuropsychological test of EFs, children are prompted to respond (by pressing a button) either to the location or the direction of an arrow appearing rapidly on the screen [57]. In the first block of the test, the rule was to judge the location of the arrow relative to a fixation point while inhibiting the direction of the point of the arrow. In the next block, subjects had to judge the direction of the arrow and inhibit the location. Each block began with a practice block of 12 trials with feedback (with a sad or a happy smiley face depending on accuracy), after which point children did not receive any feedback. Half of the stimuli were congruent trials (i.e., the location on the screen and the direction of the arrow matched), and half of them were incongruent (i.e., the location on the screen and direction of the arrow were the opposite), presented in a pseudorandom order. Both blocks had 60 trials. Figure S1 demonstrates the timing of the task. The number of correct responses in each block and the reaction time (RT) were metrics of inhibitory performance. Responses in less than 0.25 s were excluded for being too fast to be considered a response to the stimulus.

#### 2.3.2. Hearts and Flowers Test

This task was a computer-based measure of cognitive inhibition and flexibility [32,58]. In the first congruent blocks of the test, rapidly appearing hearts were presented on the left or right side of the screen. The task was to press a predetermined button on the same side of the keyboard. The aim of this block was to “warm up”; thus, it was not considered to load on executive functions. In the next incongruent block, red flowers were presented on the left or right side of the screen, and the task was to press a button on the opposite side of the keyboard. This block required inhibitory control, meaning that the prepotent tendency to respond toward a stimulus had to be inhibited for a successful trial. In the final mixed block, hearts (congruent) and flowers (incongruent) were displayed on each of the screens, and the task was to switch between the two previously learned rules and press either on the same (i.e., hearts) or the opposite side (i.e., flowers). This part required cognitive flexibility to switch between rules and inhibit incongruent trials. One stimulus was presented per trial in all blocks. Each test block was preceded by instructions and followed by 10 practice trials with feedback (smiley face) after each key response. The timing of the task is presented in Figure S1. Data were gathered regarding RT of correct answers and accuracy (number of errors in congruent and incongruent trials). Based on the protocol of Diamond and Wright [59], responses in less than 0.25 s were considered too fast to be interpreted as a response to the stimulus and were thus excluded.

#### 2.3.3. Adapted Stop Signal Task (SST)

SST measures inhibitory control [60,61,62]. In the implemented adapted SST, visual go stimuli to which a simple button press was required were infrequently followed by a subsequent visual stop stimulus that signaled that the prepotent response had to be withheld. Specifically, the go stimulus was a picture of a lion or bird, and participants had to press the L button when the lion was shown and the A button if the bird was shown (see Figure S1). Participants were requested to respond as fast and as accurately as possible to the go stimuli. The stop stimulus was a picture of a bee, and it required participants to withhold the prepotent response. For each trial, the stimuli were presented centrally and sequentially, and the stimulus duration was set at 150 ms. The trial duration was 1500 ms. The task started with a practice block, which consisted of 64 go trials. The experimental block consisted of 128 trials, of which 32 trials (25%) were stop trials. This block started with a go-stop stimulus onset asynchrony of 350 ms. Subsequently, the time between go stimulus onset and stop stimulus onset was dynamically adjusted using a tracking algorithm to yield an inhibition success of approximately 50%. The trials were randomized for each participant. The relevant outcome of the SST was the stop signal reaction time (SSRT), a measure that is thought to reflect inhibitory control. The SSRT was calculated with an integration method using the inhibition rate together with the reaction time distribution on go stimuli and the average go–stop stimulus interval [62]. A shorter SSRT corresponds to better inhibition. The mean response time was calculated by subtracting the mean RT of congruent trials from the mean RT of incongruent trials, with smaller costs indicating better performance. The percentage of correct go stimuli responses (neutral), no go stimuli inhibitions, and omissions to go stimuli were also calculated. In the present sample of children, the tracking algorithm and associated corrections of go-stop stimulus onset asynchrony yielded an inhibition rate of approximately 50%, validating our implementation of the paradigm.

#### 2.3.4. Trail Making Test (TMT)

This paper-and-pencil test is a neuropsychological measure of visual scanning, attention, and cognitive flexibility [63,64]. Task A was to connect fifteen numbers in circles from 1 to 15 with a pencil in ascending order following a numerical sequence as fast as possible (to measure visual processing speed). Task B was to connect fifteen numbers and letters in ascending order alternately following a numerical and alphabetical sequence, to assess cognitive flexibility (e.g., A-2, 2-B, B-3, etc.). Before each block, there was a practice page with a few circles. During the completion, if the examinee made a mistake, the examiner immediately stopped the examinee, pointed to the last correct circle, and asked the examinee to proceed from that point, which contributed to the overall completion time recorded.

#### 2.3.5. Resting-State Electroencephalography (EEG) Recording

Resting-state EEG measurements pre- and post-test were performed with a 14-channel Emotiv Epoc+ EEG headband, and data were transferred to an Asus X556U laptop through CyKIT 3.0 (Python server) and OpenVIBE 2.2 software [65]. The EEG data were sampled at 128 Hz from 14 electrodes placed at AF3, AF4, F3, F4, F7, F8, FC5, FC6, T7, T8, P7, P8, O1, and O2 (subset of the 10/10 system) and referenced to linked P3 and P4. However, half of the electrode positions were excluded from the final analysis (i.e., F7, T7, T8, P7, P8, O1, and O2) because the EEG data were invaluable in those positions due to calibration problems or extremely noisy data. It is important to note that the circumstances within the school context were not similar to those in the laboratory.

Raw data from the EEG measurements were processed using Python in JupyterLab. Emotiv Epoc+ automatically bandpass filtered the EEG at 0.2–45 Hz and applied a notch filter at 50 Hz. Additionally, the EEG data were corrected for DC drift with the whole segment baseline correction of each epoch. The EEG data were segmented into 2 s epochs. The epochs were baseline corrected using the average amplitude in the given epoch. Epochs with artifacts, ±75 µV deviation from the baseline, and epochs with low to no activity (all samples in the given epoch < 5 µV) were discarded. For a similar approach, see Schönenberg and colleagues [66]. With respect to dealing with eye-movement-related artifacts, a strict approach was used that involved the exclusion of those trials that included EOG blink activity, because Zeng and Song [67] reported that correction procedures, such as independent component analysis, cannot fully correct for nonstationary EOG artifacts in the EEG. The remaining nonoverlapping epochs were used to estimate power spectra using Bartlett’s method. Finally, the absolute power of theta, alpha, beta, and gamma was estimated using composite Simpson’s rule. The variables were averaged from all recorded channels to define the global absolute power of theta, alpha, and beta activity. All data (including EFs and feasibility) were analyzed with IBM SPSS Statistics (for Windows version 20.0) software [68].

#### 2.3.6. Electroencephalography (EEG) Recording during Mindfulness Sessions

Data collection and EEG-feedback of brain states during the mindfulness sessions was implemented using a 4-channel Muse brain-sensing headband and application (version 18.6). The Muse headband had four dry electrodes located at AF7, AF8, TP9, and TP10 referenced to Fpz. The data were transferred to an Android device through an application that processed raw EEG and provided the following metrics: (i) percentage of calm/focused states during a session; (ii) percentage of neutral states during a session; (iii) percentage of active/mind-wandering states during a session; (iv) number of birds during a session (referring to deep sustained focus); and (v) number of stars during a session (referring to the regulation of mind-wandering). However, to our knowledge, there is no existing research on the reliability and validity of these metrics for measuring mindfulness-related skills or performance.

### 2.4. Mindfulness Training with a Brain-Sensing Device

Based on the findings of Gruzelier [27] regarding optimal neurofeedback dosage and the mindfulness-based stress reduction protocol by Kabat-Zinn [24], this intervention consisted of 8 sessions. As displayed in the protocol, each session began with an instruction supporting the comprehension and learning of breathing awareness (see Table S1). From the third session, we shortened the instruction, given that children were already familiar with it, and added the explanation of other essential elements of mindfulness, such as the observation of sensations during breathing (e.g., through the nose tips, in the abdomen, anchors) or the acceptance and non-judgmental attitude toward the constantly changing nature of awareness (i.e., concentration level, mind-wandering). These explanations were designed in an age-appropriate language by using metaphors from previous MBIs to communicate difficult concepts [69].

The length of the sessions was gradually incremented in the following way: (a) the first and second sessions lasted for 1 min; (b) the third and fourth sessions lasted for 2 min; (c) the fifth and sixth sessions lasted for 3 min; and (d) the seventh and eighth sessions lasted for 4 min. This was recommended by Jennings and colleagues [70], who suggested shorter (3–5 min) periods of practice for children during the primary grades as they begin.

The Muse application provided a calibration period prior to each session to customize EEG-feedback to the participant’s actual state of mind with a machine learning algorithm. In the calibration period, children were asked to sit with their eyes open and then their eyes closed for a couple of minutes. During each session, the children individually practiced mindful breathing with the Muse headband and smartphone application with the experimenter in the room to provide support if necessary. The children were instructed by the experimenter to concentrate on their breathing and try to calm down the sound of the rain and hear the birds singing through the headphones. More specifically, the rain sound rumbled when the participant’s mind wandered and beta or gamma brainwaves were dominant; meanwhile, the sound of the rain turned down when attention was focused on breathing and alpha power increased [48]. Children heard birds twittering as a reward when the most desired deep meditative theta brainwaves increased in power. Each session ended with sharing and explaining the Muse application’s metrics and figures to the child (i.e., calm/focused, neutral, active/mind-wandering) and inviting the child to reflect on his/her subjective experience.

### 2.5. Procedure

Before and after the 8 sessions of mindfulness with EEG-feedback, we assessed the EFs and brain activity of all participants. Data collection took place in the rooms of the participating elementary school. The children were taken out of their class for the sessions individually. The research assistant informed children about the examination process, and the children could ask questions. After the children’s verbal consent to the examination, an approximately 30 min individual testing session began. First, resting-state neural activity was measured. Specifically, children were asked to sit calm for 150 s with their eyes open and 150 s with their eyes closed, while their brain activity was recorded with a 14-electrode wireless EEG headband (Emotiv Epoc+) on a laptop. Subsequently, the children were requested to perform four neurocognitive tests measuring EFs in a counterbalanced order. After the children were assigned to the conditions, the mindfulness group started the intervention in school as an extracurricular activity. The children were invited to individual mindfulness sessions during school hours. There was an agreement with the teacher of both classes that the children could leave their pre-arranged classes individually for 5–10 min in order to participate in the sessions. During the mindfulness sessions, neural activity was recorded with the Muse headband, and the feasibility checklist was filled out for each child. The order of the neurocognitive tests was the same for the pre- and post-test for each child. The children were rewarded with a certificate for their participation in the research project at the first meeting, and they could collect stickers on it for each task during the pre- and post-testing sessions. A few days after the pretests, the mindfulness training with EEG-feedback began. Post-testing was conducted 3 to 5 days after the last mindfulness session, and it followed the same protocol as the pretesting.

### 2.6. Statistical Analyses

All statistical analyses were conducted using SPSS 20. Prior to randomization, baseline independent sample *t*-tests were conducted to match equivalent pairs based on gender, age, and aggregated EF test scores.

To reveal any differences in changes between the mindfulness with EEG-feedback and the control group on cognitive tests and resting-state brain activity, repeated measures ANOVA tests were performed. Repeated measures ANOVAs were used in several previous studies investigating the efficacy of interventions with relatively small sample sizes [71,72]. The measurement points (two levels: pre-test and post-test) were used as a within-subject factor, while the conditions (two levels: experimental and control) were used as a between-subject factor. The assumptions to conduct repeated measures ANOVAs were tested as suggested [73,74,75]. The assumption of normality in both conditions was tested by calculating the standardized values of skewness and kurtosis in the case of all variables, as well as the significance of the Kolmogorov–Smirnov test [74]. Additionally, extreme values were identified with box plot diagrams for all variables. Values were considered extreme when they were lower or greater than the interquartile range multiplied by 1.5, based on the classic 1.5 × IQR rule [76]. Leys and colleagues [77] pointed out that unusual values that are extremely far from the central tendency can be dealt with using two statistical strategies: keeping them or removing them from the dataset. As many of the variables were non-normally distributed because of the extreme outliers, we applied these aforementioned two statistical strategies to control for this. To this end, in the first statistical model, the extreme values in non-normally distributed variables were kept. As Blanca and colleagues [78] tested, ANOVAs remain a valid statistical procedure under non-normality in a variety of conditions. The second assumption of ANOVAs, homogeneity of variances, was examined using Levene’s test, and was violated in the case of almost all variables. Although it is a controversial issue in statistics, some studies state that ANOVAs can be robust for this assumption as well [79]. In the second statistical model, we removed extreme outliers as potentially non-representative data. Regarding the nature of the outliers, we suspect that some children might have misunderstood some rules on the EF tests, which could cause an unusually high number of errors in some cases. In the case of EEG variables, we suspect that there was a great individual variability in this small sample which would be plausibly reduced in a larger sample. By removing the extreme outliers, the homogeneity of variances assumption was fulfilled for all EF variables, yet still not for EEG variables. Finally, for significant time × group interactions, exploratory paired *t*-tests were conducted to maximize insight into the relationships between variables with significant interactions, and correlation analyses were planned.

## 3. Results

### 3.1. Tests of Baseline Differences between Groups and Missing Data

Table 1 demonstrates the means and standard deviations of all EF and EEG variables. The baseline differences between groups were evaluated using one-way analysis of variance (ANOVA), and an independent sample t-test in the case of age. Between-group statistical comparison at baseline confirmed that the mean age in months (M_exp_ = 119.4, SD_exp_ = 4.99; M_contr_ = 118.7, SD_contr_ = 3.77) did not differ significantly between the mindfulness and control groups (t(29) = 0.450, *p* = 0.66). However, there was a significant baseline difference between the number of correct responses during the location block of the Stroop-like arrows test (F_(1,21)_ = 4.750, *p* = 0.04). As Table 2 shows, most of the main group effects regarding EF tests and resting-state EEG power were also non-significant (*p* > 0.05), except for the response time of the SST. It is important to note that baseline differences could challenge the interpretation of post-intervention differences between groups. However, by including time as a within-subjects factor that includes the pre-test level, and testing the time × group interaction, we effectively controlled for baseline differences.

Information about missing or excluded data is reported in Table S2. Missing data were caused by either a logging error issue in computer-based EF pretests or absence from school at post-test. The average data loss of the resting-state EEG measurement varied between 22 and 58% depending on the electrode site. Data loss was as expected and was due to the rejection of epochs consisting of artifacts, noise, muscle activity, and eye movements. In some cases (*N* = 7), EEG data were excluded because of a very low number of retained segments (<5) due to a high noise ratio.

### 3.2. Effects of Mindfulness Practice with a Brain-Sensing Device on Executive Functions and Resting-State Brain Activity

In our first statistical model, there was only one significant difference in pre- to posttest change in the mindfulness group relative to the control group, reflected by a marginally significant time × group interaction regarding the RT during the mixed block of the hearts and flowers test (see Table S3). Paired *t* tests indicated a significant decrease in mean RT for the mixed block in the mindfulness group (*t*(14) = 5.643, *p* < 0.001) and also the control group (*t*(13) = 5.708, *p* < 0.001). All other time × group interactions were non-significant, and the observed power of the tests was low. However, after exclusion of participants with significant outlying values (see statistical model 2), we found that the mindfulness group outperformed the control group on several measures. Specifically, on two EF tests, accuracy improved significantly more in the mindfulness group than in the control group. With respect to the ‘location’ block of the Stroop-like arrow test, the mindfulness group showed an increased number of correct responses reflected by a significant time × group interaction (see Table 2), with a significant growth of correct responses in the mindfulness with EEG-feedback group (*t*(11) = −4.732, *p* < 0.001) and no change in the control group (*t*(10) = −1.004, *p* = 0.34). As shown in Table 2, there was a significant time × group interaction in the ‘flower’ block of the hearts and flowers test, with a significantly greater decrease in errors in the mindfulness group relative to the control. Paired *t* tests revealed a significant decrease in errors in the mindfulness group (*t*(11) = 2.872, *p* = 0.02) and a non-significant increase in the control group (*t*(12) = −0.433, *p* = 0.67). Additionally, in the ‘flowers’ block of the hearts and flowers test, there was a marginally significant difference between the groups in the pre- to post-test change of reaction time, reflected by a time × group interaction (see Table 2), with the mindfulness group exhibiting a less decreased mean reaction time compared to the control group. Post hoc tests showed a significant decrease in RT during the flower block in the mindfulness group (*t*(12) = 5.198, *p* < 0.001) and a significant decrease in the control group (*t*(11) = 6.178, *p* < 0.001).

Importantly, these effects were mirrored by the effects on the relevant frequency bands. The results from the resting-state brain activity measurements suggested that there was an overall decline in frequency band power from pre- to post-test in both groups, except for the eyes-open alpha and theta activity in the mindfulness group. Namely, in the resting-state eyes-open condition, the analysis of variance showed a significant time × group interaction effect of the changes in theta and alpha absolute power (see Table 2). Paired *t* tests demonstrated that the control group showed a significant decrease in theta (*t*(9) = 2.458, *p* = 0.04) and alpha (*t*(9) = 2.564, *p* = 0.03), while the mindfulness group showed a non-significant change from pre- to post-test for both theta (*t*(9) = −1.073, *p* = 0.31) and alpha (*t*(9) = −0.174, *p* = 0.89). All other time × group interactions of the EF tests and EEG were non-significant (*p* > 0.05).

The exploratory correlation analysis showed a significant positive relationship between the change in RT for the flower block in the hearts and flowers test and the change in resting-state eyes-open theta activity (*r*(18) = 0.54, *p* = 0.03). There was also a marginally significant positive correlation between the change in RT for the flower block and the change in alpha activity in the eyes-open condition (*r*(18) = 0.47, *p* = 0.06). However, the correlation between the change in accuracy in the EF tests and the change in alpha or theta activity in the eyes-open condition was non-significant (*p* > 0.05). The correlation between resting-state alpha and theta activity change was positive and significant, *r*(20) = 0.92, *p* < 0.001.

### 3.3. Effects on Brain States across Mindfulness Sessions

One-way repeated measures ANOVAs were performed to analyze the modulation of three brain states (calm/focused, neutral, and active/mind-wandering) within the mindfulness with EEG-feedback group. The means were calculated for all metrics from two sessions within the same week. Thus, there were four time conditions: (1) the first and second sessions (week 1); (2) the third and fourth sessions (week 2); (3) the fifth and sixth sessions (week 3); and (4) the seventh and eighth sessions (week 4) (see Table 3).

The statistical analysis of within-subject variance showed a non-significant effect of time on the percentage of calm/focused brain states (*F*_(3, 13)_ = 2.466, *p* = 0.08, *η*_p_^2^ = 0.150); however, there was a significant linear contrast of time within the four sessions (*F*_(1, 14)_ = 5.671, *p* = 0.03, *η*_p_^2^ = 0.288). Additionally, there was a main effect of time regarding the number of birds, reflecting longer periods of time in a calm/focused brain state (*F*_(3, 13)_ = 3.200, *p* = 0.03, *η*_p_^2^ = 0.186), with a significant linear within-subjects contrast of time (*F*_(1, 14)_ = 7.489, *p* = 0.02, *η*_p_^2^ = 0.349). In summary, these results suggest a steady increase in the calm/focused brain state across the sessions.

Regarding neutral brain state, the ANOVA test also showed a significant effect of time, (*F*_(3, 13)_ = 2.09, *p* = 0.05, *η*_p_^2^ = 0.172), with a significant linear within-subjects contrast (*F*_(1, 14)_ = 7.869, *p* = 0.01, *η*_p_^2^ = 0.360).

Finally, ANOVAs of the active/mind-wandering brain states indicated a non-significant effect of time (*F*_(3, 13)_ = 0.130, *p* = 0.85, *η*_p_^2^ = 0.009) and regarding the linear within-subjects contrast, (*F*_(1, 14)_ = 0.004, *p* = 0.95, *η*_p_^2^ = 0.001). On the other hand, the number of recovery stars from the active/mind-wandering state showed a significant effect of time (*F*_(3, 13)_ = 22.959, *p* < 0.001, *η*_p_^2^ = 0.621), and the tests indicated a significant linear within-subjects contrast (*F*_(1, 14)_ = 52.069, *p* < 0.001, *η*_p_^2^ = 0.788), with an average increase in recovering from active/mind-wandering states (see Table 3).

## 4. Discussion

To the best of our knowledge, this is the first exploratory study to test the effects of an EEG-feedback-based mindfulness program on children’s executive functioning and attention-related brain activity. The aim of the present study was to explore the potential effects of a mindfulness program with EEG-feedback adopted for elementary school children to empower their own attention regulation (required for self-regulated learning), objectively measured using neurocognitive tests and brain activity.

With our first statistical model, the results showed that both groups became faster at the mixed block of the hearts and flowers test; however, the control group became faster than the other group. There were no other observed effects on the other EF variables or resting-state brain activity when we compared the mindfulness with EEG-feedback group to the control group. With the second statistical model, extreme outlier values that were potentially non-representative were excluded based on the 1.5 × IQR rule. This second statistical model provided some initial support for a potential positive effect of mindfulness training with EEG-feedback for children. Potential positive effects were found for two out of the four EF tests regarding the accuracy of inhibition- and attention-related responses. More specifically, in the hearts and flowers test, the mindfulness group made significantly fewer errors (failed inhibitions) from pre- to post-test, which was accompanied by a tendency of a decrease in reaction time from pre- to post-test. It is important to note that the control group showed an even larger decrease in RT from pre- to post-test; however, this was not accompanied by fewer errors. The improvement in RT in both groups could be attributed to a retest effect, as previous studies have shown that individuals can improve due to experience with the tasks or other non-specific factors [80]; however, the errors changed differently in the two groups. As Diamond [81] described, errors are often made because of not being able to wait; if inhibition is well-developed, errors can be avoided. These findings may suggest that mindfulness training with EEG-feedback empowered children to regulate their immediate responses and slow down, which at least contributed to the enhanced inhibitory performance. Furthermore, the mindfulness group showed significantly more correct responses (successful inhibitions) on the Stroop-like arrow test from pre- to post-test compared to the control group. This effect was not accompanied by any changes in RT. A recent meta-analysis by Sumantry and Stewart [82] concluded that mindfulness led to greater improvements in accuracy-based tasks rather than reaction time, which is in line with our findings. Interestingly, in the study of Bhayee and colleagues [16] with adults, reaction time (RT) results showed a somewhat different effect: the neurofeedback-assisted mindfulness group’s inhibitory responses on the Stroop task became faster, while their accuracy did not change. As Davidson and colleagues [32] concluded, inhibition requires greater effort from children, which can be seen in the errors of difficult trials, while RT remains relatively constant, in contrast to adults whose RT slows down in difficult trials. Our findings extend this literature by showing a change in accuracy (related to inhibition) due to mindfulness, with a preliminary effect on RT, similar to the above-mentioned adult performance.

The results from the resting-state brain activity measurement suggested that in the eyes-open condition, the mindfulness with EEG-feedback group showed no change in alpha and theta absolute power from pre- to post-test, while the control group showed a significant decrease in these low-frequency neural oscillations. To fully understand alpha and theta neural oscillations among children, it is important to note that longitudinal research has demonstrated that infant EEG is at a much lower frequency, which increases with aging [83]. For instance, in relaxed wakefulness when the alpha frequency from 8 Hz to 13 Hz is dominant in adults, infants exhibit a lower frequency range from 6 Hz to 9 Hz [84]. Therefore, it could be that the participating 9- to 10-year-old children in our study also exhibited a somewhat lower frequency range for the alpha band than adults, and the observed increase in the theta band could demonstrate an increase in the alpha band. To connect our neuropsychological findings to previous research, we concluded that the non-significant increase in baseline alpha and theta oscillations in the mindfulness with EEG-feedback group accompanied by significant improvement in inhibition and attention was somewhat surprising, given that both neurofeedback and mindfulness separately were found to increase these brain waves in previous studies [27,35]. Interestingly, Navarro-Gil and colleagues [51] also found that baseline alpha was not modulated by alpha upregulating neurofeedback training, and only task-related alpha increased.

Finally, an exploratory correlation analysis showed that the pre-to-post changes in resting-state eyes-open theta and alpha activity were positively correlated with the changes in RT in the executive function test (where accuracy increased in the mindfulness with EEG-feedback group). These findings lend support to the theory of Klimesch and colleagues [41], who proposed that alpha oscillations have two central roles in information processing, namely timing and inhibition. Our exploratory results extend Klimesch’s theory by showing that an increase in baseline alpha oscillations (before the EF task) was associated with improved information processing speed during an EF task and thus timing. However, it is important to note that we could not inspect whether alpha during the task was associated with timing or inhibition because there was no EEG measurement during the EF task.

The results from the second exploratory analysis showed that there was a linear effect of time regarding the percentage of time spent in a calm/focused state during mindfulness with EEG-feedback sessions. From the first to the last session, children improved by 8% on average at being in a calm/focused state during the session. Additionally, there was a significant linear effect of time regarding the longer and deeper focused states (birds/minute), showing 1.5 birds/minute more on average during the last session than in the first. These indicators, measured by the Muse headband and application, suggest an increase in the lower frequency alpha and theta brain waves during the training and thus a relaxed yet focused mind state. The variance of analysis regarding the change of neutral states during the technology-supported mindfulness training also indicated a linear effect of time, with a 9% mean decrease from the first session to the last session. Interestingly, the mean active/mind-wandering states were generally very low even during the first session among the sample (1% of the session duration), and this stayed true for the last session as well. However, the mean number of recovery stars per minute showed a significant increase from the first to the last session, with a mean of 5.8 recovery stars per minute increase, which suggests that the children improved in recognizing mind-wandering states and redirecting their attention to their breathing. These results are somewhat contradictory to the findings of Acabchuk and colleagues [13], who found non-significant changes in the calm/focused states of adults in the Muse group from pre- to post-test. This non-significant difference between the pre- and post-test may be due to high individual variance in mindfulness performance within sessions, with decreases, stagnation, and increases in performance over the whole training. Our exploratory results extend prior research by highlighting the potential to investigate the learning process from session to session instead of only focusing on pre- and post-test measures of mindfulness. It may also raise attention to the need for repeated measures of state mindfulness due to the high variance between different mindfulness sessions.

### Limitations

There were five perceived limitations in this pilot study: (i) low sample size; (ii) a passive control group; (iii) a low-intensity training protocol; (iv) the lack of blinding of conditions; and (v) the possibility of a carryover effect. We addressed the first limitation in the current study by conducting ANOVAs, which are quite robust and are claimed to be applicable to relatively low sample sizes [85]. However, as the power analysis showed, the sample size was underpowered to detect small effects. Regarding the methodological limitations of the study, we highly recommend adding an active control condition to the design (i.e., sham-feedback or mindfulness group) to rule out non-specific (e.g., training or placebo) effects, from effects specific to the supplementation of EEG-feedback to simple mindfulness. In addition, we cannot exclude the presence of expectancy bias from the experimenters and participants due to the lack of blinding of conditions; thus, this could also be addressed in future studies. Another possible confounding effect could be observed in the study from the carryover effect of mindfulness practice on post-test resting-state EEG measurement, as the results showed a clear reduction in alpha and theta activity in the control group from pre-to-post but not in the mindfulness group. We applied the post-test EEG measurement 3–5 days after the last mindfulness session; however, future studies could aim to test the effects at a more delayed post-test or at follow-up. In subsequent research, the carryover effect might be addressed by counterbalancing the order of EEG measurement and cognitive tests, or by planning resting-state EEG measurement as the last measurement after cognitive tests to avoid the sequential order of mindfulness practice and resting-state EEG measurement [86].

Another limitation that is also important to note is that the variables obtained from the Muse headset (brain states, birds, and stars) are derived from black-box algorithms, which brings their reliability and validity into question. The study of Kovacevic and colleagues [48] provided some information about the outline of the algorithms; however, important details were not reported. This lack of a clear EEG-feedback protocol might also be addressed in future research by applying a predesigned protocol (i.e., alpha and theta training); for more examples see [27,34].

Moreover, another perceived limitation was that our sample consisted of a restricted age and SES range (9–10 years, middle and high SES). Hence, an applicable nuance should be applied when generalizing these results to other samples. Furthermore, we did not control for demographic characteristics in the statistical analyses (i.e., SES, intelligence, etc.) which could also influence the cognitive outcomes in children, and the statistical analyses were not corrected for multiple comparisons.

## 5. Conclusions

Based on our findings, it can be concluded that mindfulness training with EEG-feedback (provided by the Muse headband) linearly increased calm/focused brain states and the redirection of attention when it wandered. With our first line of statistical models, no positive effects on executive functions and resting-state brain activity could be observed when we compared the mindfulness and control groups. However, with the second more stringent line of statistical models excluding outliers, significant changes from pre- to post-test were detected for two out of the four EF tasks and in the resting-state eyes-open alpha and theta brain activity between the mindfulness and the control group. More specifically, the mindfulness with EEG-feedback group showed a significant improvement in inhibition and information processing compared to the control group. Our findings extend Klimesch’s [41] theory by connecting baseline alpha brain activity with information processing during a cognitive task. These findings provide some preliminary evidence for technology-supported mindfulness practice embedded in everyday practice in schools to empower children to practice regulating their own attention without the assistance of an adult. The results from our pilot study also call attention to future research with a larger sample, a longer intervention, and a sham-feedback group.

## Figures and Tables

**Table 1:** Means and standard deviations of all dependent variables included in the study.

Dependent Variable	*n*	Pre *M* (*SD*)	*n*	Post *M* (*SD*)	*n*	Pre *M* (*SD*)	*n*	Post *M* (*SD*)
Mindfulness					Control			
**Executive function measures**								
*Hearts and flowers test*								
Flowers block RT	13	0.55 (0.07)	13	0.50 (0.06)	12	0.55 (0.08)	12	0.48 (0.12)
Mixed block RT	15	0.85 (0.09)	15	0.75 (0.07)	10	0.90 (0.08)	10	0.73 (0.14)
Flowers block errors	12	1.92 (1.51)	12	0.92 (0.90)	13	1.46 (1.27)	13	2.00 (2.00)
Mixed block errors	14	5.29 (3.65)	14	4.50 (2.79)	12	5.42 (3.97)	12	4.86 (3.28)
*Location–direction Stroop-like arrows test*								
Location block RT	15	0.54 (0.04)	15	0.53 (0.07)	15	0.50 (0.06)	15	0.49 (0.07)
Direction block RT	15	0.61 (0.04)	15	0.61 (0.04)	11	0.60 (0.03)	11	0.59 (0.04)
Location block correct responses	12	48 (7.97)	12	55 (5.78)	11	54 (4.71)	11	56 (2.48)
Direction block correct responses	15	25 (10.4)	15	38 (13.4)	15	30 (14.9)	15	41 (11.2)
*Stop signal task*								
SSRT	10	321 (67.7)	10	274 (58.2)	11	389 (192)	11	262 (82.3)
Response time	9	838 (116)	9	772 (125)	11	618 (195)	11	713 (177)
% of omissions	10	4.79 (4.40)	10	1.77 (2.87)	8	6.38 (3.67)	8	2.47 (3.10)
*Trail making test (B)*								
Errors	13	0.38 (0.65)	13	0.08 (0.28)	14	0.71 (0.99)	14	0.29 (0.61)
Completion time	13	46.9 (15.7)	13	39.9 (22.6)	15	53.4 (21.8)	15	38.8 (14.7)
**EEG measures**								
*Resting-state eyes-closed condition—Global mean absolute power* (µ*V*^2^)								
Theta	9	3.69 (3.51)	9	3.58 (3.63)	9	5.56 (15.81)	9	2.18 (1.50)
Alpha	9	2.45 (3.32)	9	2.67 (3.12)	9	3.10 (2.06)	9	1.30 (0.84)
Beta	10	5.48 (4.01)	10	4.20 (3.99)	8	5.86 (1.82)	8	4.80 (3.96)
*Resting-state eyes-open condition—Global mean absolute power* (µ*V*^2^)								
Theta	10	2.92 (2.70)	10	3.69 (3.36)	10	5.21 (3.56)	10	2.19 (1.14)
Alpha	10	1.65 (1.71)	10	1.69 (1.79)	10	2.52 (1.37)	10	1.13 (0.49)
Beta	12	6.93 (5.27)	12	5.26 (4.72)	9	8.83 (4.73)	9	4.60 (3.37)

*M*, mean; *RT*, reaction time in milliseconds (ms); *SST*, stop signal task; *SSRT*, stop signal reaction time in milliseconds (ms); *TMT*, trail making test; *completion time* in seconds (s); frequencies were fixed for *theta* (4–8 Hz), *alpha* (8–12 Hz), and *beta* (12–30 Hz).

**Table 2:** Results of the repeated measures ANOVAs (model 2).

Dependent Variable	Time	Group	Time × Group
***Hearts and flowers test***			
Flowers block RT	*F*_(1, 23)_ = 66.347, *η*_p_^2^ = 0.743 **	*F*_(1, 23)_ = 0.256, *η*_p_^2^ = 0.011	*F*_(1, 23)_ = 3.847, *η*_p_^2^ = 0.143 ^+^
Mixed block RT	*F*_(1, 23)_ = 65.404, *η*_p_^2^ = 0.740 **	*F*_(1, 23)_ = 1.847, *η*_p_^2^ = 0.074	*F*_(1, 23)_ = 1.485, *η*_p_^2^ = 0.061
Flowers block errors	*F*_(1, 23)_ = 2.879, *η*_p_^2^ = 0.111 *	*F*_(1, 23)_ = 0.071, ηp ^2^ = 0.003	*F*_(1, 23)_ = 5.353, *η*_p_^2^ = 0.189 *
Mixed block errors	*F*_(1, 24)_ = 3.645, *η*_p_^2^ = 0.190	*F*_(1, 24)_ = 0.016, ηp ^2^ = 0.002	*F*_(1, 24)_ = 0.243, *η*_p_^2^ = 0.022
***Location–direction Stroop-like arrows test***			
Location block RT	*F*_(1, 28)_ = 1.379, *η*_p_^2^ = 0.047	*F*_(1, 28)_ = 2.844, *η*_p_^2^ = 0.090	*F*_(1, 28)_ = 0.003, *η*_p_^2^ = 0.001
Direction block RT	*F*_(1, 24)_ = 0.033, *η*_p_^2^ = 0.001	*F*_(1, 24)_ = 1.923, *η*_p_^2^ = 0.074	*F*_(1, 24)_ = 0.345, *η*_p_^2^ = 0.014
Location block correct responses	*F*_(1, 21)_ = 14.917, *η*_p_^2^ = 0.415 **	*F*_(1, 21)_ = 2.943, *η*_p_^2^ = 0.123	*F*_(1, 21)_ = 5.433, *η*_p_^2^ = 0.206 *
Direction block correct responses	*F*_(1, 28)_ = 35.856, *η*_p_^2^ = 0.562 **	*F*_(1, 28)_ = 1.026, *η*_p_^2^ = 0.035	*F*_(1, 28)_ = 0.221, *η*_p_^2^ = 0.008
***Stop signal task***			
SSRT	*F*_(1, 19)_ = 6.944, *η*_p_^2^ = 0.268 *	*F*_(1, 19)_ = 0.543, *η*_p_^2^ = 0.028	*F*_(1, 19)_ = 1.454, *η*_p_^2^ = 0.071
Response time	*F*_(1, 18)_ = 0.040, *η*_p_^2^ = 0.002	*F*_(1, 18)_ = 5.023, *η*_p_^2^ = 0.218 *	*F*_(1, 18)_ = 4.291, *η*_p_^2^ = 0.193 *
% of omissions	*F*_(1, 16)_ = 11.984, *η*_p_^2^ = 0.428 *	*F*_(1, 16)_ = 0.699, *η*_p_^2^ = 0.042	*F*_(1, 16)_ = 0.196, *η*_p_^2^ = 0.012
*Trail making test*			
Errors	*F*_(1, 25)_ = 5.020, *η*_p_^2^ = 0.167 *	*F*_(1, 25)_ = 1.672, *η*_p_^2^ = 0.063	*F*_(1, 25)_ = 0.135, *η*_p_^2^ = 0.005
Completion time	*F*_(1, 26)_ = 8.867, *η*_p_^2^ = 0.254 *	*F*_(1, 26)_ = 0.192, *η*_p_^2^ = 0.007	*F*_(1, 26)_ = 1.069, *η*_p_^2^ = 0.040
***Resting-state eyes-closed condition*** ***—Global mean absolute power*** **(** **µ** ***V*** **^2^** **)**			
Theta	*F*_(1, 16)_ = 2.613, η *η*_p_^2^ = 0.140	*F*_(1, 16)_ = 0.033, *η*_p_^2^ = 0.002	*F*_(1, 16)_ = 2.311, *η*_p_^2^ = 0.126
Alpha	*F*_(1, 16)_ = 1.963, *η*_p_^2^ = 0.109	*F*_(1, 16)_ = 0.118, *η*_p_^2^ = 0.007	*F*_(1, 16)_ = 3.241, *η*_p_^2^ = 0.168
Beta	*F*_(1, 16)_ = 0.083, *η*_p_^2^ = 0.063	*F*_(1, 16)_ = 0.142, *η*_p_^2^ = 0.009	*F*_(1, 16)_ = 0.010, *η*_p_^2^ = 0.001
***Resting-state eyes-open condition*** ***—Global mean absolute power*** **(** **µ** ***V*** **^2^** **)**			
Theta	*F*_(1, 18)_ = 2.500, *η*_p_^2^ = 0.122	*F*_(1, 18)_ = 0.452, *η*_p_^2^ = 0.022	*F*_(1, 18)_ = 7.093, *η*_p_^2^ = 0.283 *
Alpha	*F*_(1, 18)_ = 5.135, *η*_p_^2^ = 0.222 *	*F*_(1, 18)_ = 0.073, *η*_p_^2^ = 0.004	*F*_(1, 18)_ = 5.804, *η*_p_^2^ = 0.244 *
Beta	*F*_(1, 19)_ = 6.369, *η*_p_^2^ = 0.251 *	*F*_(1, 19)_ = 0.135, *η*_p_^2^ = 0.007	*F*_(1, 19)_ = 1.197, *η*_p_^2^ = 0.059

*RT*, reaction time in milliseconds (ms); *SST*, stop signal task; *SSRT*, stop signal reaction time in milliseconds (ms); *TMT*, trail making test; *completion time* in seconds (s); *η*_p_^2^, partial eta squared effect size; effect size (*η*_p_^2^*)* interpreted as: small—0.01, medium—0.06, large—0.14; ^+^ *p* < 0.06; * *p* < 0.05; ** *p* < 0.001. Average differences across the two groups and the corresponding 95% confidence interval were calculated with a standard 0.05 significance level (two-tailed).

**Table 3:** Within-group changes in brain states during the mindfulness sessions with EEG-feedback (*n* = 15).

	Session 1 and 2	Session 3 and 4	Session 5 and 6	Session 7 and 8
	Mean (SD)			
Calm/focused state (%)	60 (21.65)	56 (18.79)	67 (19.48)	68 (23.70)
Neutral state (%)	39 (20.77)	43 (17.99)	32 (18.19)	30 (21.94)
Active/mind-wandering state (%)	1 (3.03)	2 (3.23)	2 (2.57)	1 (1.99)
Birds/minute	4.5 (3.44)	3.9 (2.65)	5.6 (2.84)	6.0 (3.04)
Stars/minute	0.2 (0.42)	3.9 (2.65)	5.6 (2.84)	6.0 (3.03)

## Data Availability

The datasets analyzed in this study can be found in the OSF Repository [https://osf.io/3amw8/?view_only=7402d0534f9a47cc92fa097dc71a6d25]. (Last accessed date: 27 November 2021).

## Supplementary Materials

The following are available online at https://www.mdpi.com/article/10.3390/brainsci12010103/s1, Figure S1: Timing of the computer-based neuropsychology tasks, Table S1: The content and protocol of the mindfulness program with the Muse brain-sensing device for school-aged children, Table S2: Significant statistical outliers and missing data in the statistical models, Table S3: Results of the ANOVAs on EFs and brain activity in statistical model 1.
