# Supplementary material for: Mindfulness Practice with a Brain-Sensing Device Improved Cognitive Functioning of Elementary School Children: An Exploratory Pilot Study
Source: Brain Sci. 2022 Jan 12;12(1):103. doi: 10.3390/brainsci12010103 (PMC8774020; doi:10.3390/brainsci12010103)
Supplement: Supplementary file 1 [file brainsci-12-00103-s001.zip › brainsci-1505779-supplementary.pdf]

Figure S1. Timing of the computer-based neuropsychology tasks

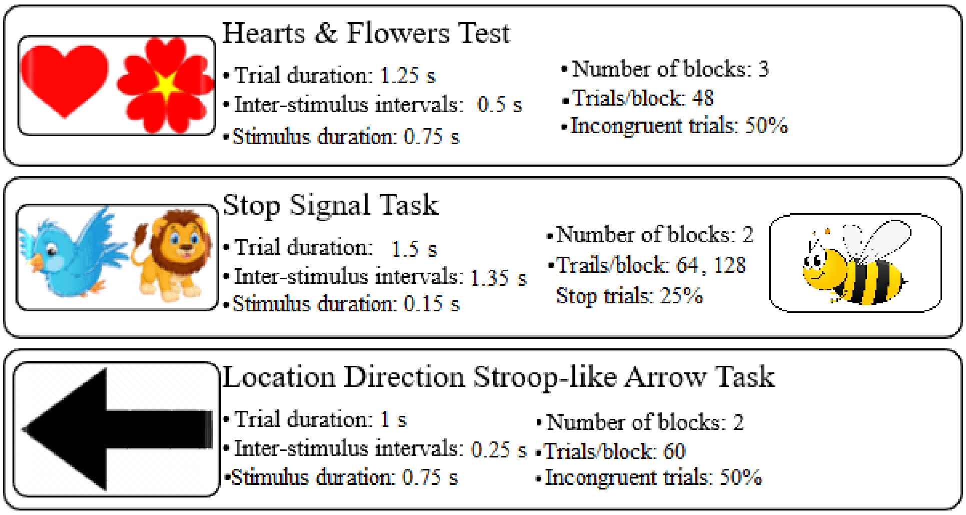

Table S1. The content and protocol of the mindfulness program with the Muse brain-sensing device for school-aged children

Session	Objective	Activities
1	Introduction of the Muse Interaxon headband, and the neurofeedback system	• Conversation about attention with/without awareness, and the nature of attention with the puppy metaphor<br>• Breathing awareness meditation<br>• Conversation about the child's current state of attention based on the Muse application results
2	Children will be able to understand the neurofeedback system, and practice how to guide their attention to the breathing	• Reminder: repetition of the instruction<br>• Breathing awareness meditation<br>• Conversation about the child's current state of attention based on the Muse application results
3	Children will be able to notice the body sensations connected to breathing, and use them as an anchor of attentional awareness	• Tips & Tricks: body sensation during inhalation and exhalation, and how to use them as an anchor for awareness<br>• Breathing awareness meditation<br>• Conversation about the child's current state of attention based on the Muse application results
4	Children will be able to use body sensation as anchors to breathing awareness, and to intentionally pay attention to breathing	• Reminder: repetition of bodily sensations as anchors of attention<br>• Breathing awareness meditation<br>• Conversation about the child's current state of attention based on the Muse application results

5	Children will be able to notice their current state of attention, and accept it without judgement or evaluation	• Conversation about the changing nature of attention (e.g., concentration, mind-wandering) and accepting as it is<br>• Breathing awareness meditation<br>• Conversation about the child's current state of attention based on the Muse application results
6	Children will be able to accept their current state of attention, and develop a kind non-judgemental attitude towards themselves	• Reminder: repetition of acceptance and non-judgemental attitude towards our own current state<br>• Breathing awareness meditation<br>• Conversation about the child's current state of attention based on the Muse application results
7	Children will be able to focus their attention for a longer period of time	• Breathing awareness meditation<br>• Conversation about the child's current state of attention based on the Muse application results
8	Children will be able to master the skills required for intentionally paying attention to their own breathing for a longer period of time	• Breathing awareness meditation<br>• Conversation about the child's current state of attention based on the Muse application results

Table S2. Significant statistical outliers and missing data in the statistical models

**Statistical model no. 1**	**Missing data (n)**
Location Direction Stroop-like Arrow test	absence from school (1)
Hearts & Flowers test	logging error (1)
SST	failure to estimate the SSRT, or Mean RT, or omissions due to incorrect switching of finger-stimulus assignment (10-13)
TMT	logging error (1)
Resting-state eyes-closed brain activity	<5 retained segments of EEG data due to high noise ratio (7)
Resting-state eyes-open brain activity	<5 retained segments of EEG data due to high noise ratio (6)
**Statistical model no. 2**	**Excluded significant statistical outliers (n)**
Location Direction Stroop-like Arrow test	direction block mean RT (5), location block correct responses (8)
Hearts & Flowers test	flowers block mean RT (4), flowers block errors (4), mixed block mean RT (4), mixed block errors (3)
TMT	errors (3), completion time (2)
Resting-state eyes-closed brain activity	theta & alpha (6), beta (6)
Resting-state eyes-open brain activity	theta & alpha (2), beta (1)

TMT B Trail Making Test B version

Table S3. Results of the ANOVAs on EFs and brain activity in statistical model no. 1

	**Statistical model No.1**		
**Dependent variable**	**Time**	**Group**	**Time × group**
Hearts & Flowers test			
Flowers block RT	$F_{(1, 27)}$ = 14.533, η_p^2 = .350**	$F_{(1, 27)}$ = 0.106, η_p^2 = .004	$F_{(1, 27)}$ = 0.167, η_p^2 = .006
Mixed block RT	$F_{(1, 27)}$ = 62.936, η_p^2 = .700**	$F_{(1, 27)}$ = 0.044, η_p^2 = .002	$F_{(1, 27)}$ = 3.852, η_p^2 = .125$^+$
Flowers block errors	$F_{(1, 27)}$ = 0.180, η_p^2 = .007	$F_{(1, 27)}$ = 0.306, η_p^2 = .011	$F_{(1, 27)}$ = 0.979, η_p^2 = .035
Mixed block errors	$F_{(1, 27)}$ = 6.331, η_p^2 = .190*	$F_{(1, 27)}$ = 0.180, η_p^2 = .007	$F_{(1, 27)}$ = 0.614, η_p^2 = .022
Location Direction Stroop-like Arrows test			
Location block RT	$F_{(1, 28)}$ = 1.379, η_p^2 = .047	$F_{(1, 28)}$ = 2.844, η_p^2 = .090	$F_{(1, 28)}$ = 0.003, η_p^2 = .001
Direction block RT	$F_{(1, 28)}$ = 0.513, η_p^2 = .018	$F_{(1, 28)}$ = 6.777, η_p^2 = .195*	$F_{(1, 28)}$ = 0.101, η_p^2 = .0004
Location block correct responses	$F_{(1, 28)}$ = 14.511, η_p^2 = .341 **	$F_{(1, 28)}$ = 1.469, η_p^2 = .050	$F_{(1, 28)}$ = 0.001, η_p^2 = .001
Direction block correct responses	$F_{(1, 28)}$ = 35.856, η_p^2 = .562 **	$F_{(1, 28)}$ = 1.026, η_p^2 = .035	$F_{(1, 28)}$ = 0.221, η_p^2 = .008
Stop Signal Task			
SSRT	$F_{(1, 19)}$ = 6.944, η_p^2 = .268*	F $_{(1, 19)}$ = 0.543, η_p^2 = 0.028	$F_{(1, 19)}$ = 1.454, η_p^2 = .071
Response Time	$F_{(1, 19)}$ = 0.156, η_p^2 = .008	F $_{(1, 19)}$ = 3.996, η_p^2 = 0.174 $^+$	$F_{(1, 19)}$ = 1.599, η_p^2 = .078
% of omissions	$F_{(1, 19)}$ = 0.267, η_p^2 = .014	F $_{(1, 19)}$ = 3.308, η_p^2 = 0.148	$F_{(1, 19)}$ = 1.412, η_p^2 = .069
Trail Making Test			
Errors	$F_{(1, 26)}$ = 1.145, η_p^2 = .041	$F_{(1, 26)}$ = 1.482, η_p^2 = .052	$F_{(1, 26)}$ = 0.105, η_p^2 = .040
Completion time	$F_{(1, 26)}$ = 8.867, η_p^2 = .254*	$F_{(1, 26)}$ = 0.192, η_p^2 = .007	$F_{(1, 26)}$ = 1.069, η_p^2 = .040
Resting-state EEG measurement			
Theta (eyes-closed)	$F_{(1, 19)}$ = 4.575, η_p^2 = .194*	F $_{(1, 19)}$ = 0.097, η_p^2 = 0.005	$F_{(1, 19)}$ = 0.582, η_p^2 = .030
Alpha (eyes-closed)	$F_{(1, 19)}$ = 3.442, η_p^2 = .153	F $_{(1, 19)}$ = 0.070, η_p^2 = 0.004	$F_{(1, 19)}$ = 0.553, η_p^2 = .028
Beta (eyes-closed)	$F_{(1, 19)}$ = 3.565, η_p^2 = .158	F $_{(1, 19)}$ = 1.137, η_p^2 = 0.156	$F_{(1, 19)}$ = 0.902, η_p^2 = .045
Theta (eyes-open)	$F_{(1, 20)}$ = 3.943, η_p^2 = .165$^+$	F $_{(1, 20)}$ = 0.408, η_p^2 = 0.020	$F_{(1, 20)}$ = 0.277, η_p^2 = .014
Alpha (eyes-open)	$F_{(1, 20)}$ = 5.164, η_p^2 = .205*	F $_{(1, 20)}$ = 0.452, η_p^2 = 0.022	$F_{(1, 20)}$ = 0.052, η_p^2 = .003
Beta (eyes-open)	$F_{(1, 20)}$ = 6.718, η_p^2 = .251*	F $_{(1, 20)}$ = 0.889, η_p^2 = 0.043	$F_{(1, 20)}$ = 2.530, η_p^2 = .112

RT reaction time in milliseconds (ms); *SST* Stop Signal Task; *SSRT* Stop Signal Reaction Time in milliseconds (ms); *TMT* Trail Making Test; *Completion time* in seconds (s); ηp^2 partial eta squared effect size; effect size (η_p^2) interpreted as :small – 0.01, medium – 0.06, large – 0.14; $^+$ $p < 0.06$; *$p < 0.05$; **$p < 0.001$.
